# Effects of Different Types of Exercise Training on the Cortisol Awakening Response in Children

**DOI:** 10.3389/fendo.2019.00463

**Published:** 2019-07-26

**Authors:** Mirko Wegner, Flora Koutsandréou, Anett Müller-Alcazar, Franziska Lautenbach, Henning Budde

**Affiliations:** ^1^Department of Sport Psychology, Institute of Sports Science, Humboldt University Berlin, Berlin, Germany; ^2^Faculty of Human Sciences, Medical School Hamburg, Hamburg, Germany; ^3^Department of Sport Psychology, Institute for Sport Psychology and Sport Pedagogy, Leipzig University, Leipzig, Germany

**Keywords:** cortisol, exercise training, physical stress, children, adolescents, motor exercise, cardiovascular exercise

## Abstract

**Context:** Due to great variability of the hypothalamus-pituitary-adrenal (HPA)-axis, research has to produce better-controlled findings to make a more meaningful statement regarding the effect of exercise training (ET) on the cortisol awakening response (CAR), especially in children.

**Objective:** The aim of the study was to investigate the effects of different ET interventions on the CAR in children.

**Design and setting:** We conducted a short-term training study for 10 weeks in primary schools in Westphalia, Germany.

**Participants:** 71 children (9–10 years old) were randomly assigned to a cardiovascular exercise group (*n* = 27), a motor exercise group (*n* = 23), or a control group (*n* = 21).

**Intervention:** An experienced instructor trained the children in an after-school setting in 45 min sessions, three times a week over the course of 10 weeks.

**Main outcome measure:** CAR (0, +30 min) was assessed on 2 schooldays one week apart before and after the 10-week intervention. A Shuttle Run Test was performed to determine the cardiovascular fitness. Motor fitness was assessed using the Heidelberg Gross Motor Test.

**Results:** Children who enhanced their cardiovascular fitness over the course of the intervention showed an increased CAR after the intervention time (*B* = 0.213), whereas children who underwent a motor exercise intervention and at the same time gained in motor fitness exhibited a decreased CAR after intervention (*B* = −0.188).

**Conclusions:** It has been speculated that other neurobiological pathways are activated by different exercise interventions. The extent to which these ET effects on CAR can be applied in clinical settings needs further investigation.

**Précis:** The 10-weeks longitudinal effects of cardiovascular vs. motor exercise interventions (three times a week) on CAR in children show that these interventions exert different effects on hypothalamus-pituitary-adrenal (HPA) axis activity.

## Introduction

Children and adolescents in industrialized countries today increasingly suffer from inactivity and associated health issues such as obesity and psychological disorders, e.g., as an effect of stress exposure (1). Exercise training (ET), however, is an easy-to-implement intervention, which can be administered in group settings including schools. It has been proven that ET has long-term beneficial effects and is a cost-efficient and sustainable strategy to improve health in various mental and physical disorders (2, 3). ET is defined as a structured exercise program that involves the use of large muscle groups for extended periods of time. ET differs from physical activity (PA) in its planned and structured nature (4). Even though it appears that experts believe prevention of diseases should start in childhood and adolescence, there are rarely studies that focus on the effects of ET on health in children under the age of 12 (5). The present study focuses on an age group that lacks extensive research on stress related hormonal indices (e.g., HPA axis activity).

The HPA axis is a highly stress-responsive system and shows a strong diurnal pattern with the glucocorticoid cortisol as an end product. One suitable marker for determining HPA axis activity is the CAR. The CAR is a reliable measure for the acute responsiveness of the HPA axis and can serve as a useful index of adrenocortical activity (6). Cortisol levels, which are measured during the first 30 min after awakening show an increase of 50–70% in the vast majority of adults but generally seem to be less pronounced in children and adolescence (7). In adults, CAR is generally positively associated with job and general life stress (8). Even though it is still unclear what contributes to a “healthy” CAR, ET has been argued to alter the HPA axis activity in adults depending on the intensity of the intervention (e.g., moderate vs. vigorous) as well as on the intervention type (e.g., aerobic vs. yoga) (6). Thus, exercise intensity is an issue like in other interventions to promote mental health (9, 10).

To our knowledge, there are no randomized and controlled intervention studies focusing on the effects of ET on the CAR in children. Only a few studies investigated the relationship between ET and CAR in children and adolescents in cross-sectional designs presenting insconsistent results. For example, a positive correlation between CAR and vigorous PA was found in a study focusing on 8-year-old girls suffering from metabolic syndrome (11). In contrast, a lower CAR was linked to the duration of acute daytime sport among healthy older adolescents (aged 10–18 years) (12). Finally, a study among healthy 8-year-old children showed no differences in the diurnal salivary cortisol pattern based on the level of the overall daytime PA (13). However, children's general physical fitness level and regular physical activity were not assessed, which could affect cortisol activity. Further, these were all cross-sectional study designs, which limit causal relationships between ET/PA and CAR.

As the exercise intervention type (6) might affect chronic stress levels (e.g., CAR), it could be argued that exercise interventions that focus on improvement of motor abilities might be beneficial for preserving cognitive resources and thus, freeing resources to deal with complex situations in daily life and resulting in a reduced stress response (14). Cardiovascular exercise, by contrast, may lead to stronger neurogenesis, which might result in stronger cortisol responses after a chronic exercise intervention (15). It was previously argued that hippocampal neurogenesis is mainly promoted by cardiovascular exercise interventions (16). Niemann et al. (17) could not reveal changes in hippocampal volume after 6 months of motor demanding training, but found a significant increase in volume after cardiovascular training in elderlies. Thus, specific stressors influence neurogenesis and the HPA axis activity in different ways (18).

Overall, the existing literature does not allow for causal inference because it is unknown if the parameter itself (being more physically fit) caused the changes in HPA axis activity or if other factors might have accounted for these differences. Therefore, controlled intervention studies are required to focus on the causal relationship between exercise and HPA axis activity (e.g., CAR) in a young, pre-adolescent age group. Martikainen et al. (13) stipulated that the exercise intervention type needs to be manipulated. Taken together, the current study aims to fill this gap by investigating the effect of a 10-week ET intervention (cardiovascular vs. motor fitness group) on the HPA axis activity in 8- to 10-year-old children. We hypothesized that cardiovascular exercise leads to an increased CAR response and motor exercise training results in a decreased, or no change in, CAR response among children.

## Methods

### Participants

Data^1^ of 71 prepubescent primary school children (39 female) between 9 and 10 years (M_*age*_ = 9.4; *S*D_*age*_ = 0.6) with no psychological or physical impairments (e.g., obesity) were randomly assigned to a cardiovascular exercise group (CV, *n* = 27), a motor exercise group (MO, *n* = 23), or a control group (CON, *n* = 21).

### Inclusion/Exclusion Criteria

All participants were recruited from local schools and inclusion criteria were 9–10 years of age, right-handedness, corrected-to or normal vision and prepubescent status according to parent and self-report on the Tanner staging system (below a score of 2 on the five-point scale) (20). In case of the presence of mental and physical impairments and/or previous or actual intake of psychoactive substances, participants were deemed ineligible. Before the study commenced, the ethics committee of the German Psychological Society approved the protocol (HB 02201 6_amd_092011). All participants and their legal guardians provided informed written consent after study procedures were explained in detail. The study was conducted following the guidelines set forth in the declaration of Helsinki and registered in the German Clinical Trials Register (DRKS00016590).

### Measurements

#### Cortisol Awakening Response (CAR)

**Figure 1:**
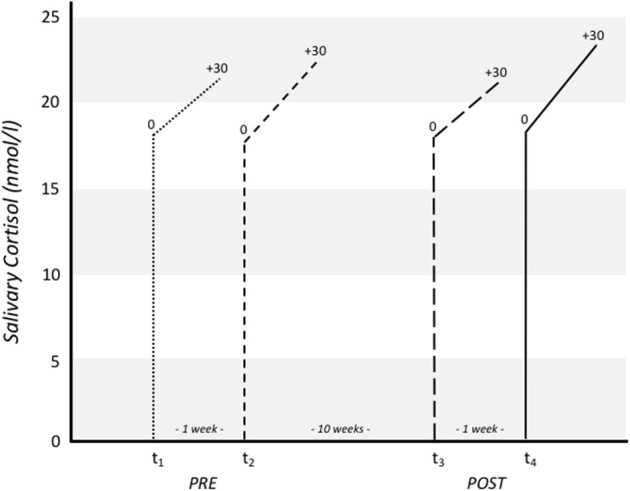
Salivary cortisol raw scores (nmol/l) for each measurement point pre (t_1_, t_2_) and post (t_3_, t_4_) intervention.

The CAR can be defined as the change in cortisol concentration immediately post-awakening: it represents a discrete aspect of the cortisol circadian cycle and has good intra-individual stability across time (21). Morning salivary cortisol levels were assessed at home at two time points. Children obtained saliva samples 0 and 30 min after awakening with the help of their parents, who were instructed personally and with the help of written manuals. Wakening time was 7 o'clock for all children. In order to increase reliability of the measure two cortisol samples (0, +30 min) were taken one week later both on the same day of the week pre- and post-intervention. Children were asked to refrain from physical activity one day before the assessment. Overall salivary cortisol was sampled eight times (see Figure 1 and Table 1). Saliva samples were analyzed from whole saliva collected via the SaliCap®system (IBL, Hamburg, Germany). For each assessment, participants were asked to accumulate saliva in their mouth for 2 min and refrain from swallowing while doing so. The accumulated saliva was then transferred into a pre-labeled vial via a straw. After arriving at school, research staff collected and stored the samples at −20°C until analysis. Cortisol levels (nmol/l) were analyzed using a commercially available enzyme-linked immunoassay (IBL, Hamburg, Germany) at the Biochemical Laboratory of the Department of Clinical Biopsychology, University of Marburg. Intra- and inter-assay coefficients of variation were 6.7 and 7.6%, respectively.

**Table 1:** Salivary cortisol raw scores (nmol/l) for each time point.

	**Minutes from awakening**	
	**0**	**30**
**PRE**		
t1	17.99 (7.77)	21.39 (9.68)
t2	17.73 (10.82)	22.24 (8.95)
**POST**		
t3	17.98 (8.90)	21.34 (12.50)
t4	18.27 (8.30)	23.29 (12.60)

CAR was calculated as the area under the curve (AUCg), in a first step using the mean of both saliva samples (immediately and 30 min after awakening) for pre and post-intervention separately, as it has been shown that awakening cortisol levels might be sensitive to differences in daily activities. In a final step, AUCg was calculated in accordance to Fekedulegn et al. (22). The raw scores for salivary cortisol are illustrated in Figure 1.

#### Cardiovascular Fitness (Card Fit)

Card Fit was tested with the Shuttle Run Test, a standard method for determining cardiorespiratory fitness in school children. Children were asked to run between two lines set 20 m apart. In accordance with the standardization used elsewhere (23), the children ran back and forth continuously with an initial speed of 8.0 km.h-1, increasing the level by 0.5 km h^−1^ each minute. Acoustic signals in a given frequency were used to control the pace. In all stages the students were motivated by cheering and by a pacemaker. We determined the HR_max_ as well as the maximum scores reached, which are the level and number of shuttles reached before fatigue (i.e., unable to maintain pace).

#### Motor Fitness (Mot Fit)

Mot Fit was assessed using the Heidelberg Gross Motor Test for children. We included the performance of six motor tasks [i.e., balance, rhythm, spatiotemporal orientation, and motor adaptation to moving objects; for further details see Koutsandréou et al. (19)] that were quantitatively measurable and calculated a sum score. For example, in the motor adaptation to moving objects task, points were earned by first throwing a ball backword through straddled legs against a 3 m distant wall and then, catching the rebounding ball (two points) or just touching or dropping it (one point).

#### Intervention

For 10 weeks, three times a week, for 45 min, an experienced exercise instructor trained the participants after school in groups of 7–14 children. The CV group trained their cardiovascular fitness via running and running-based games, however, varied to avoid boredom. The MO group focused on improving fine and gross motor body coordination through playful coordination exercises with low intensity: for the cardiovascular system. The control group received assisted homework sessions to prevent attention bias and control for retest effects (19). As previously reported (19) the three experimental groups (MO: 125.4 bpm, CV: 138.8 bpm, CON: 79.4 bpm) differed significantly regarding their mean heart rate levels: (recorded by F1 Polar HR monitors; Polar, Kempele, Finland) during exericse with MO and CV scoring significantly higher than CON but also CV scoring slightly higher than MO.

### Procedure

For the pre-post-design of this study, cardiovascular and motor fitness were assessed in the week prior to the start of the intervention, and 1 week before the last intervention appointment. Following the recommendation by Hellhammer et al. (24), CAR was assessed twice prior and after the intervention: 1 week prior (1st time pre) and on the day of the start of the intervention (2nd time pre), as well as 1 week before the last intervention appointment and at the end of the 10-week intervention.

### Statistical Analyses

For statistical analysis cortisol values were log-transformed to achieve normal distribution. A series of hierarchical regression analyses were performed in three blocks to predict post-intervention average CAR value for the two assessments (t_3_, t_4_), and to control for lower order effects before testing for higher order effects (see Table 1). In the first block we controlled for sex, the average value of CAR for the two pre-intervention assessments (t_1_, t_2_), pre-intervention cardiovascular fitness level, and age. In the second block, the experimental groups (MO vs. CON, CV vs. CON) were included as categorical variables in the model, as well as the change scores for cardiovascular and motor fitness. In the third block, the interaction terms between cardiovascular/motor fitness and intervention condition were added (cardiovascular fitness × CV; cardiovascular fitness × MO; motor fitness × CV; motor fitness × MO). For statistical analysis SPSS 24 software (IBM, Armonk, USA) was used.

## Results

**Figure 2:**
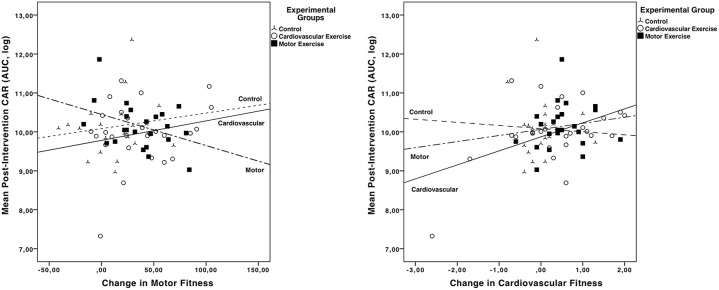
Post-intervention CARs (log-transformed AUC) in the three experimental groups (CV, MO, Control) as function of change in motor fitness (left graph) and change in cardiovascular fitness (right graph).

In order to test the effects of a 10-week physical exercise intervention on motor vs. cardiovascular fitness on CAR in children, we computed a hierarchical regression analysis using three blocks (see Table 2). In the first block, post-intervention CAR was residualized for age, gender, pre-cardiovascular performance, and pre-intervention CAR. Including pre-intervention CAR (*B* = 0.284, *p* = 0.001), age (*B* = 0.094, ns), gender (*B* = −0.170, ns), and pre-cardiovascular performance (*B* = −0.012, ns) rendered the regression analysis on post-intervention CAR significant, R^2^ = 0.187, *F*_(4, 65)_ = 3.730, *p* = 0.009. In Model 2, we included changes in participants' intervention-induced cardiovascular (ΔCard Fit) and motor fitness (ΔMot Fit), as well as participants' training intervention [cardiovascular exercise training (CV) or motor exercise training group (MO)] compared to the control group in the model. In Model 3, the interaction terms (multiplicative term of z-standardized variables) of training intervention (CV vs. MO) by changes in participants' fitness levels (ΔMot Fit, ΔCard Fit) were added to the regression. Adding the cardiovascular and motor exercise group and the cardiovascular and motor pre-to-post-fitness changes did not result in a significant improvement in Model 2. However, including the interaction terms significantly improved Model 3, ΔR^2^ = 0.130, Δ*F*_(4, 57)_ = 2.914, *p* = 0.029, and rendered the whole model significant, R^2^ = 0.366, *F*_(12, 57)_ = 2.353, *p* = 0.005 (see Table 2). Participants in the CV who enhanced their cardiovascular fitness over the course of the intervention showed an increased CAR after the intervention time (*B* = 0.213), whereas children who underwent a motor exercise intervention and at the same time gained in motor fitness exhibited a decreased CAR after 10 weeks of intervention (*B* = −0.188; see Figure 2).

**Table 2:** Results of the hierarchical regression analysis of post-intervention CAR (cortisol awakening response, log-transformed AUC) with gender, age, pre-intervention CAR, and performance in the shuttle run test (Pre CP) were entered in Model 1.

**Variable**	**Post-CAR**											
	**Model 1**				**Model 2**				**Model 3**			
	***B***	***SE***	***t***	***p***	***B***	***SE***	***t***	***p***	***B***	***SE***	***t***	***p***
Constant	6.716	1.447	4.639	0.000	6.919	1.455	4.754	0.000	7.026	1.416	4.962	0.000
Gender	−0.170	0.160	−1.059	0.293	−0.154	0.164	−0.940	0.351	−0.226	0.162	−1.394	0.169
Pre-CAR	0.284	0.079	3.585	0.001	0.295	0.080	3.666	0.001	0.271	0.077	3.506	0.001
Pre-CP	−0.012	0.045	−0.259	0.796	0.018	0.049	0.371	0.712	0.045	0.047	0.950	0.346
Age	0.094	0.133	0.703	0.485	0.040	0.136	0.292	0.771	0.049	0.132	0.372	0.711
CE					−0.031	0.096	−0.323	0.748	−0.017	0.091	−0.191	0.849
ME					−0.009	0.096	−0.094	0.925	−0.003	0.090	−0.033	0.974
ΔCard Fit					0.167	0.087	1.924	0.059	0.215	0.083	2.572	0.013
ΔMot Fit					0.013	0.082	0.164	0.870	0.032	0.078	0.414	0.680
CE × ΔCard Fit									0.213	0.099	2.157	0.035
ME × ΔCard Fit									0.072	0.100	0.720	0.474
CE × ΔMot Fit									0.018	0.093	0.189	0.851
ME × ΔMot Fit									−0.188	0.093	−2.016	0.049
*R^2^*	0.187				0.236				0.366			
*F*	3.73				2.35				2.74			
*df*	(4.65)				(8.61)				(12.57)			
Δ*R^2^*					0.049				0.130			
Δ*F*					0.981				2.914			
*df*					(4.61)				(4.57)			

*PreCP, Pre-cordiocosculor performance. Experimental group assignment (cardiovascular exercise group, CE, and motor exercise group, ME), and increases in cardiovascular fitness (ΔCardFit) and motor fitness (ΔMotFit) were entered in Model 2. Lastly, interaction terms between variables were entered in Model 3*.

## Discussion

The aim of this study was to investigate the effects of cardiovascular vs. motor exercise interventions on CAR in 9 to 10-year-old children. Our results show that cardiovascular and motor exercise exert different effects on HPA axis activity. Thus, specific exercises influence the HPA axis activity in different ways. Whereas, an increase in cardiovascular fitness was accompanied by an increase in HPA axis activity, particularly in children who underwent a cardiovascular exercise program, an increase in motor fitness in children who underwent a motor exercise program was accompanied by a decrease in CAR.

First, it should be kept in mind that it is still challenging to identify whether a large or small CAR is considered “healthy.” Second, to our knowledge, there is not enough systematic research on children, who represent an under-studied population. Research in this field is scarce, which makes it challenging to put results into context and highlights the importance of addressing the effects of different exercise interventions on the cortisol activity in children in future research. We base our argumentation on findings in adults showing that a higher increase in CAR is generally positively associated with job and general life stress (8).

Children who did increase their cardiovascular fitness, regardless of their experimental group assignment, showed an increase in cortisol activity. This is in line with previous findings in children indicating vigorous physical activity was positively related with 30 min post-waking cortisol values (11). With respect to children that did increase their cardiovascular fitness and presented an increase in cortisol activity, it could be argued that this is a result of an HPA axis hyper-responsiveness as a biological consequence of the frequent activation of the axis triggered by exercise stress: this may be part of the physiological adaptation of the neuroendocrine system to chronic demands (25).

Utilizing a set of salivary cortisol data in a small sample population of healthy older adults (mean age 65), a robust cortisol awakening response, and increased CAR after exercise training (a 6-month supervised intervention designed to reach 60–70% of their maximum heart rate reserve, 3 days a week, without any mentioned motor demands) were observed (26). However, one has to keep in mind that the cortisol response like many mental health related responses to exercise is depending on age (27).

Children in the motor exercise group that did increase their motor fitness showed a decrease in cortisol activity. This result is in line with research linking lower CAR to the duration of daytime sport (12). In a study with healthy young adults, the impact of long duration and high intensity of exam stress on the CAR supports that the HPA axis is down-regulated by chronic major stress, with this downregulation reflected by a reduction of the CAR (28). Although the mechanisms resulting in hypocortisolism are not yet fully clarified, possible explanations include changes in the biosynthesis of HPA-axis hormones and/or availability and functioning of their receptors at all levels of the HPA-axis [see Heim et al. (29) for discussion].

One could argue that due to improved motor abilities cognitive resources are freed to deal with complex situations in daily life resulting in a reduced chronic stress response (14). It has been further suggested that having control or no control over stress can have opposite effects on neural plasticity (15). Regarding the training of the cognitive system in this group one could speculate that it leads to an improved self-regulation and thus influencing neural input/traffic to the HPA axis. Results by Blair et al. (30) indicate that moderately higher levels of cortisol are associated with better performance on self-regulation, however it remains to be evaluated if this elevation in cortisol would lead to a change in CAR. One could also argue that the cognitive challenge of this exercise should induce neurogenesis (31). However, these argumentations would impede an explanation of the observed differences. Only the following hypothesis backed up by human data can somehow explain our findings. A recent study about the hippocampal volume in older humans after a 6-month intervention period, indicates that only, cardiovascular but not motor demanding training led to increases in hippocampal volume (17) which is positively related to the magnitude of the CAR (32).

One limitation of the study is that we only used two time points to assess the AUCg for the CAR. However, this procedure has been previously presented by different authors (5). Another limitation is that in the motor exercise group it is not possible to standardize the intensity for the neural nor for the cardiovascular system to compensate for motor demands of the different exercises, which can be challenging for one participant and more difficult for another participant. It cannot be ruled out that training programs matched for cardiovascular load, but with different coordinative demands would have resulted in a different pattern of results. As we previously reported both experimental exercise groups differed in intensity (19, 33).

Also, even though the children in the MO group were provided with playful exercises, and children in the CV group completed a variation of running exercises, it remains unclear how they were perceived by the children themselves. Finally, we did not control for subjectively perceived (chronic) stress prior or post-intervention. Future research, especially when focusing on children, should do so in order to control for potential confounding variables, as general life stress in adults has been associated with differences in CAR (8).

Another limitation of our study is that we did not assess abdominal fat in these children. It is known that abdominal fat may affect neuroendocrine responses mainly to psychological stress (34). However, significant changes in abdominal fat usually take place during puberty and children in our study were on average 9.4 years old which might diminish the effect of abdominal fat on HPA axis reactivity. Also, there is not enough systematic research on this specific understudied population and research in this field has not been well-discussed in the literature yet. This should be addressed in future studies and encourage researchers to add abdominal fat as a covariate.

One advantage of the present study though is that the AUCg used presents a more stable measure of the CAR because we used two AUCg measurements 1 week apart as previously recommended in the CAR guidelines by Stalder et al. (35). However, as training intensity and volume play a crucial role for the effects on CAR (6), future research should implement follow-up measurement points.

Overall, our results show that cardiovascular and motor exercise training in school exerts different effects on HPA axis activity. Whereas, an increase in cardiovascular fitness was accompanied by an increase in HPA axis activity, an increase in motor fitness in children was lead to a decrease in CAR.

Research has yet to produce more detailed and consistent findings to make a more meaningful statement regarding ET and its role on CAR, especially in children. The current study raises questions that future research needs to address in order to increase prevention of potential pathological diseases in childhood and adolescence.

Unfortunately, we simply do not know under what circumstances, for whom, and at what developmental periods under- vs. over-activation of the HPA-axis are most likely and how this is expressed by changes in CAR in this age group. While we suspect that under-activation of the HPA-axis may in fact be a reflection of more severe stress exposure and have more serious consequences than hyperactivation (36), however it needs to be established what the consequences are and under which cortisol concentration they occur.

## Ethics Statement

Before the study commenced, the ethics committee of the German Psychological Society approved the protocol (HB 02201 6_amd_092011). All participants and their legal guardians provided informed written consent after study procedures were explained in detail. The study was conducted following the guidelines set forth in the declaration of Helsinki and registered in the German Clinical Trials Register (DRKS00016590).

## Author Contributions

MW, HB, and FK contributed conception and design of the study. FK organized the database. FK and MW performed the statistical analysis. FK, MW, HB, and FL wrote the first draft of the manuscript. MW, HB, FK, FL, and AM-A wrote sections of the manuscript. All authors contributed to manuscript revision, read and approved the submitted version.

### Conflict of Interest Statement

The authors declare that the research was conducted in the absence of any commercial or financial relationships that could be construed as a potential conflict of interest.

## Footnotes

^1^Data from this sample (including working memory performance) have been previously reported (19) but neither of the analyses on HPA activity included in the current manuscript.
